# (*E*)-1-(1-Hy­droxy­naphthalen-2-yl)-3-(2,4,5-trimeth­oxy­phen­yl)prop-2-en-1-one

**DOI:** 10.1107/S1600536813006843

**Published:** 2013-03-16

**Authors:** Dongsoo Koh

**Affiliations:** a Department of Applied Chemistry, Dongduk Women’s University, Seoul 136-714, Republic of Korea

## Abstract

In the title mol­ecule, C_22_H_20_O_5_, the C=C bond of the central enone group adopts an *E* conformation. The dihedral angle formed by the benzene ring and the naphthalene ring system is 12.6 (4)°. The hy­droxy group attached to the naphthalene ring is involved in an intra­molecular O—H⋯O hydrogen bond. In the crystal, weak C—H⋯O hydrogen bonds link the mol­ecules into chains along [010]. In addition, π–π stacking inter­actions are present, with centroid–centroid distances of 3.6648 (15) and 3.8661 (15) Å between the benzene and two naphthalene rings.

## Related literature

For the synthesis and biological properties of chalcone deriv­atives, see: Shenvi *et al.* (2013 ▶); Hsieh *et al.* (2012 ▶); Sharma *et al.* (2012 ▶); Sashidhara *et al.* (2011 ▶); Aponte *et al.* (2010 ▶); Hans *et al.* (2010 ▶) Jo *et al.* (2012 ▶). For related structures, see: Park *et al.* (2013 ▶); Fadzillah *et al.* (2012 ▶). For standard bond lengths, see: Allen *et al.* (1987 ▶).
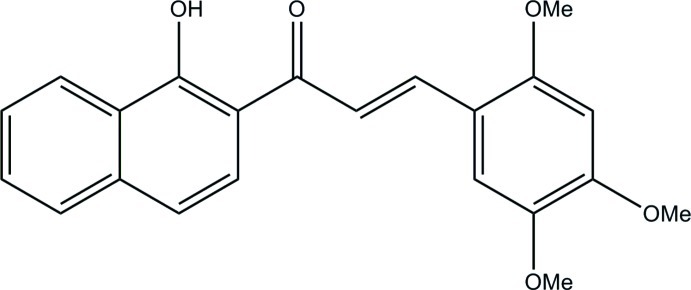


## Experimental

### 

#### Crystal data

C_22_H_20_O_5_
*M* *_r_* = 364.38
Monoclinic, 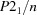

*a* = 9.7919 (12) Å
*b* = 13.7559 (18) Å
*c* = 13.2761 (17) Å
β = 96.165 (3)°
*V* = 1777.9 (4) Å^3^
*Z* = 4
Mo *K*α radiation
μ = 0.10 mm^−1^
*T* = 200 K
0.36 × 0.26 × 0.22 mm

#### Data collection

Bruker SMART CCD diffractometer
Absorption correction: multi-scan (*SADABS*; Bruker, 2000 ▶) *T*_min_ = 0.966, *T*_max_ = 0.979
12937 measured reflections
4420 independent reflections
2550 reflections with *I* > 2σ(*I*)
*R*_int_ = 0.032

#### Refinement

*R*[*F* ^2^ > 2σ(*F* ^2^)] = 0.053
*wR*(*F* ^2^) = 0.187
*S* = 1.10
4420 reflections
248 parameters
H-atom parameters constrained
Δρ_max_ = 0.31 e Å^−3^
Δρ_min_ = −0.40 e Å^−3^

### 

Data collection: *SMART* (Bruker, 2000 ▶); cell refinement: *SAINT* (Bruker, 2000 ▶); data reduction: *SAINT*; program(s) used to solve structure: *SHELXS97* (Sheldrick, 2008 ▶); program(s) used to refine structure: *SHELXL97* (Sheldrick, 2008 ▶); molecular graphics: *PLATON* (Spek, 2009 ▶); software used to prepare material for publication: *SHELXTL* (Sheldrick, 2008 ▶).

## Supplementary Material

Crystal structure: contains datablock(s) I, global. DOI: 10.1107/S1600536813006843/aa2085sup1.cif

Structure factors: contains datablock(s) I. DOI: 10.1107/S1600536813006843/aa2085Isup2.hkl

Supplementary material file. DOI: 10.1107/S1600536813006843/aa2085Isup3.cml

Additional supplementary materials:  crystallographic information; 3D view; checkCIF report

## Figures and Tables

**Table 1:** Hydrogen-bond geometry (Å, °)

*D*—H⋯*A*	*D*—H	H⋯*A*	*D*⋯*A*	*D*—H⋯*A*
O5—H5⋯O1	0.84	1.74	2.490 (2)	147
C21—H21⋯O3^i^	0.95	2.43	3.362 (3)	166

Symmetry code: (i) 
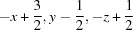
.

**Fig. 1.**
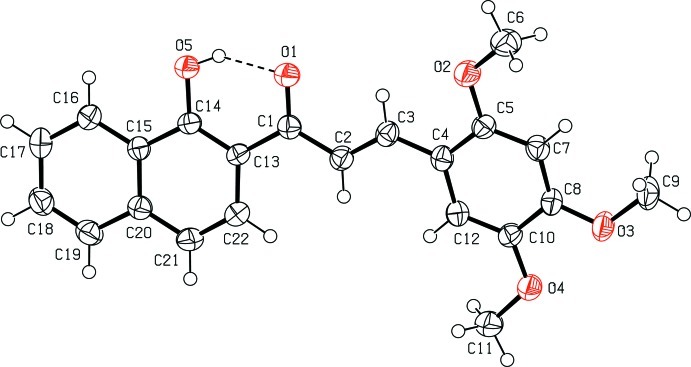
Molecular structure of the title compound showing displacement ellipsoids drawn at the 50% probability level. The dashed line indicates an intramolecular hydrogen bond.

**Fig. 2.**
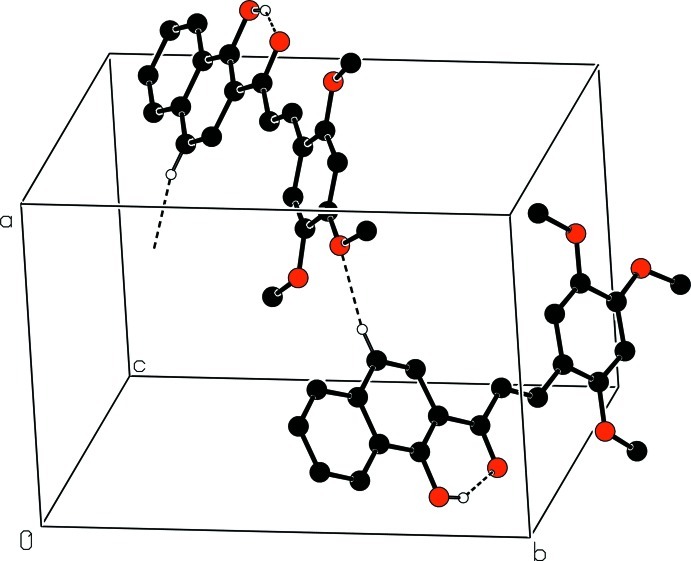
Part of the crystal structure with O—H···O and weak intermolecular C—H···O hydrogen bonds shown as dashed lines.

## supplementary crystallographic information

### Comment

Varieties of chalcones have been synthesized and isolated from natural sources, which have been used for evaluation of their pharmaceutical applications. They have showed diverse biological activities including anticancer (Shenvi *et al.* 2013), antidiabetic (Hsieh *et al.* 2012), antimicrobial (Sharma *et al.* 2012), anti-Leishmania (Aponte *et al.* 2010), anti-inflammatory (Sashidhara *et al.* 2011) and antitubercular (Hans *et al.* 2010). In continuation of our research interest to develop benzochalcone derivatives which show broad range of biological activities (Jo *et al.* 2012), titled compound was synthesized and its crystal structure was determined.

The molecular structure of the title compound is shown in Fig. 1. Chalcone is a family of flavonoid class which has a general C_6_—C_3_—C_6_ carbon framework and that of C_3_ is an α,β-unsaturated carbonyl (enone) group. One of the C_6_ is substituted with C_10_ (naphthalene ring) in the benzochalcone, where the titled compound belongs to. The C2═C3 bond of the central enone group adopts a *trans* configuration. The dihedral angle formed by the naphthalene ring system and the benzene ring is 12.6 (4)°. Due to an intramolecular O—H···O hydrogen bond between the hydroxy group of the naphthalene ring and carbonyl (C═O) group, the C1═O1 bond [1.262 (3) Å] is slightly longer than the standard value (Allen *et al.* 1987). In the crystal, weak C—H···O hydrogen bonds link the molecules into one-dimensional chains along [010] (Fig. 2). In addition, intermolecular π–π stacking interactions are present with Cg1···Cg2(1-x, 2-y, -z) = 3.6648 (15)Å and Cg1···Cg3(1-x, 2-y, 1-z) = 3.8661 (15)Å, where Cg1, Cg2 and Cg3 are the centroids of the C4/C5/C7/C8/C10/C12, C13/C14/C15/C20/C21/C22 and C15/C16/C17/C18/C19/C20 rings.

Examples of structures of substituted prop-2-en-1-one compounds have been published (Park *et al.*, 2013; Fadzillah *et al.*, 2012).

### Experimental

To a solution of 2,4,5-trimethoxybenzaldehyde (196 mg, 1 mmol) in 10 ml of ethanol was added 1-hydroxy-2-acetonaphthone (186 mg, 1 mmol) and the temperature was adjusted to around 275–276 K in an ice-bath. To the cooled reaction mixture 1 ml of 50% aqueous KOH solution was added, and the reaction mixture was stirred at room temperature for 20 h. This mixture was poured into iced water (30 ml) was acidified (pH = 3) with 6 N HCl solution to give a precipitate. Filtration and washing with water afforded crude solid of the title compound (180 mg, 48%). Recrystallization of the solid from ethanol gave yellow colored crystals (mp: 471–472 K).

### Refinement

The H atoms were placed in calculated positions with C—H = 0.95 and 0.98 Å or O—H = 0.84 Å, and refined in a riding-model approximation with *U*_iso_(H) = 1.2 *U*_eq_(C) or 1.5 *U*_eq_(C_methyl_) and *U*_iso_(H) = 1.5 *U*_eq_(O)].

### Crystal data

**Table:**

C_22_H_20_O_5_	*F*(000) = 768
*M**_r_* = 364.38	*D*_x_ = 1.361 Mg m^−^^3^
Monoclinic, *P*2_1_/*n*	Mo *K*α radiation, λ = 0.71073 Å
Hall symbol: -P 2yn	Cell parameters from 3912 reflections
*a* = 9.7919 (12) Å	θ = 2.5–28.2°
*b* = 13.7559 (18) Å	µ = 0.10 mm^−^^1^
*c* = 13.2761 (17) Å	*T* = 200 K
β = 96.165 (3)°	Block, red
*V* = 1777.9 (4) Å^3^	0.36 × 0.26 × 0.22 mm
*Z* = 4	

### Data collection

**Table:**

Bruker SMART CCD diffractometer	4420 independent reflections
Radiation source: fine-focus sealed tube	2550 reflections with *I* > 2σ(*I*)
Graphite monochromator	*R*_int_ = 0.032
φ and ω scans	θ_max_ = 28.3°, θ_min_ = 2.1°
Absorption correction: multi-scan (*SADABS*; Bruker, 2000)	*h* = −13→12
*T*_min_ = 0.966, *T*_max_ = 0.979	*k* = −17→18
12937 measured reflections	*l* = −12→17

### Refinement

**Table:**

Refinement on *F*^2^	Primary atom site location: structure-invariant direct methods
Least-squares matrix: full	Secondary atom site location: difference Fourier map
*R*[*F*^2^ > 2σ(*F*^2^)] = 0.053	Hydrogen site location: inferred from neighbouring sites
*wR*(*F*^2^) = 0.187	H-atom parameters constrained
*S* = 1.10	*w* = 1/[σ^2^(*F*_o_^2^) + (0.0702*P*)^2^ + 1.1296*P*] where *P* = (*F*_o_^2^ + 2*F*_c_^2^)/3
4420 reflections	(Δ/σ)_max_ < 0.001
248 parameters	Δρ_max_ = 0.31 e Å^−^^3^
0 restraints	Δρ_min_ = −0.40 e Å^−^^3^

### Special details

**Table:**

Geometry. All e.s.d.'s (except the e.s.d. in the dihedral angle between two l.s. planes) are estimated using the full covariance matrix. The cell e.s.d.'s are taken into account individually in the estimation of e.s.d.'s in distances, angles and torsion angles; correlations between e.s.d.'s in cell parameters are only used when they are defined by crystal symmetry. An approximate (isotropic) treatment of cell e.s.d.'s is used for estimating e.s.d.'s involving l.s. planes.
Refinement. Refinement of *F*^2^ against ALL reflections. The weighted *R*-factor *wR* and goodness of fit *S* are based on *F*^2^, conventional *R*-factors *R* are based on *F*, with *F* set to zero for negative *F*^2^. The threshold expression of *F*^2^ > σ(*F*^2^) is used only for calculating *R*-factors(gt) *etc*. and is not relevant to the choice of reflections for refinement. *R*-factors based on *F*^2^ are statistically about twice as large as those based on *F*, and *R*- factors based on ALL data will be even larger.

### Fractional atomic coordinates and isotropic or equivalent isotropic displacement parameters (Å^2^)

**Table:**

	*x*	*y*	*z*	*U*_iso_*/*U*_eq_	
C1	0.3412 (2)	0.91056 (17)	0.00657 (18)	0.0317 (5)	
O1	0.22475 (17)	0.94630 (13)	−0.02165 (13)	0.0404 (4)	
C2	0.4142 (3)	0.93980 (17)	0.10242 (18)	0.0345 (5)	
H2	0.5043	0.9158	0.1210	0.041*	
C3	0.3569 (2)	1.00024 (17)	0.16584 (18)	0.0338 (5)	
H3	0.2662	1.0212	0.1439	0.041*	
C4	0.4152 (2)	1.03710 (17)	0.26228 (18)	0.0320 (5)	
C5	0.3361 (2)	1.09604 (17)	0.32022 (18)	0.0336 (5)	
O2	0.20245 (17)	1.11129 (14)	0.27987 (14)	0.0450 (5)	
C6	0.1146 (3)	1.1618 (2)	0.3405 (2)	0.0518 (7)	
H6A	0.1132	1.1282	0.4054	0.078*	
H6B	0.0214	1.1639	0.3052	0.078*	
H6C	0.1485	1.2283	0.3525	0.078*	
C7	0.3909 (2)	1.13575 (18)	0.41125 (19)	0.0356 (6)	
H7	0.3356	1.1760	0.4486	0.043*	
C8	0.5259 (2)	1.11725 (18)	0.44823 (18)	0.0346 (5)	
O3	0.59060 (18)	1.15449 (14)	0.53450 (14)	0.0454 (5)	
C9	0.5169 (3)	1.2224 (2)	0.5899 (2)	0.0528 (8)	
H9A	0.4829	1.2757	0.5451	0.079*	
H9B	0.5782	1.2485	0.6467	0.079*	
H9C	0.4391	1.1895	0.6159	0.079*	
C10	0.6075 (2)	1.05692 (17)	0.39235 (19)	0.0341 (5)	
O4	0.74055 (18)	1.04546 (13)	0.43608 (13)	0.0437 (5)	
C11	0.8242 (3)	0.9805 (2)	0.3870 (2)	0.0468 (7)	
H11A	0.7824	0.9157	0.3840	0.070*	
H11B	0.9156	0.9773	0.4250	0.070*	
H11C	0.8323	1.0037	0.3181	0.070*	
C12	0.5528 (2)	1.01849 (17)	0.30251 (18)	0.0336 (5)	
H12	0.6084	0.9780	0.2657	0.040*	
C13	0.3995 (2)	0.83943 (16)	−0.05961 (17)	0.0286 (5)	
C14	0.3299 (2)	0.81550 (16)	−0.15212 (17)	0.0293 (5)	
O5	0.20698 (16)	0.85556 (12)	−0.18480 (13)	0.0366 (4)	
H5	0.1858	0.8966	−0.1422	0.055*	
C15	0.3832 (2)	0.74664 (17)	−0.21887 (17)	0.0306 (5)	
C16	0.3122 (3)	0.72114 (18)	−0.31235 (19)	0.0373 (6)	
H16	0.2270	0.7516	−0.3340	0.045*	
C17	0.3644 (3)	0.65246 (19)	−0.3731 (2)	0.0435 (6)	
H17	0.3151	0.6351	−0.4361	0.052*	
C18	0.4906 (3)	0.6082 (2)	−0.3416 (2)	0.0441 (6)	
H18	0.5263	0.5606	−0.3835	0.053*	
C19	0.5629 (3)	0.63260 (19)	−0.2518 (2)	0.0418 (6)	
H19	0.6491	0.6025	−0.2325	0.050*	
C20	0.5116 (2)	0.70218 (17)	−0.18647 (18)	0.0331 (5)	
C21	0.5822 (3)	0.72680 (19)	−0.0915 (2)	0.0389 (6)	
H21	0.6679	0.6969	−0.0702	0.047*	
C22	0.5289 (2)	0.79279 (18)	−0.03049 (18)	0.0361 (6)	
H22	0.5783	0.8084	0.0329	0.043*	

### Atomic displacement parameters (Å^2^)

**Table:**

	*U*^11^	*U*^22^	*U*^33^	*U*^12^	*U*^13^	*U*^23^
C1	0.0357 (13)	0.0287 (12)	0.0309 (12)	−0.0018 (10)	0.0048 (10)	0.0028 (9)
O1	0.0385 (10)	0.0434 (10)	0.0383 (10)	0.0090 (8)	−0.0004 (8)	−0.0037 (8)
C2	0.0345 (13)	0.0341 (13)	0.0343 (13)	−0.0023 (10)	0.0006 (10)	−0.0004 (10)
C3	0.0358 (12)	0.0315 (12)	0.0336 (13)	−0.0068 (10)	0.0009 (10)	0.0007 (10)
C4	0.0352 (12)	0.0284 (12)	0.0326 (12)	−0.0061 (9)	0.0044 (10)	0.0005 (9)
C5	0.0302 (12)	0.0354 (13)	0.0349 (13)	−0.0018 (10)	0.0022 (10)	−0.0005 (10)
O2	0.0339 (9)	0.0566 (12)	0.0439 (11)	0.0061 (8)	0.0016 (8)	−0.0103 (9)
C6	0.0406 (15)	0.067 (2)	0.0478 (16)	0.0113 (14)	0.0061 (13)	−0.0046 (15)
C7	0.0331 (12)	0.0386 (13)	0.0360 (13)	0.0004 (10)	0.0081 (10)	−0.0055 (10)
C8	0.0353 (13)	0.0353 (13)	0.0332 (13)	−0.0057 (10)	0.0033 (10)	−0.0069 (10)
O3	0.0399 (10)	0.0559 (12)	0.0397 (10)	−0.0011 (9)	0.0007 (8)	−0.0197 (9)
C9	0.0534 (17)	0.0592 (18)	0.0459 (16)	−0.0023 (14)	0.0053 (13)	−0.0252 (14)
C10	0.0292 (12)	0.0365 (13)	0.0361 (13)	−0.0016 (10)	0.0011 (10)	−0.0015 (10)
O4	0.0367 (10)	0.0513 (11)	0.0419 (10)	0.0065 (8)	−0.0013 (8)	−0.0126 (8)
C11	0.0413 (14)	0.0516 (17)	0.0468 (16)	0.0090 (13)	0.0008 (12)	−0.0044 (13)
C12	0.0351 (12)	0.0319 (12)	0.0339 (13)	−0.0026 (10)	0.0049 (10)	−0.0021 (10)
C13	0.0261 (11)	0.0303 (11)	0.0289 (12)	−0.0017 (9)	0.0006 (9)	0.0032 (9)
C14	0.0266 (11)	0.0270 (11)	0.0337 (12)	−0.0021 (9)	0.0008 (9)	0.0041 (9)
O5	0.0327 (9)	0.0403 (10)	0.0361 (9)	0.0061 (7)	0.0004 (7)	−0.0037 (7)
C15	0.0304 (12)	0.0290 (12)	0.0326 (12)	−0.0027 (9)	0.0036 (9)	0.0005 (9)
C16	0.0351 (13)	0.0382 (14)	0.0376 (14)	−0.0025 (10)	−0.0001 (10)	−0.0033 (11)
C17	0.0514 (16)	0.0403 (15)	0.0387 (14)	−0.0050 (12)	0.0043 (12)	−0.0099 (11)
C18	0.0446 (15)	0.0415 (15)	0.0467 (16)	0.0035 (12)	0.0083 (12)	−0.0089 (12)
C19	0.0380 (14)	0.0378 (14)	0.0499 (16)	0.0058 (11)	0.0061 (12)	−0.0014 (12)
C20	0.0326 (12)	0.0306 (12)	0.0363 (13)	−0.0013 (10)	0.0047 (10)	0.0019 (10)
C21	0.0309 (12)	0.0419 (14)	0.0432 (14)	0.0042 (11)	0.0000 (11)	0.0009 (11)
C22	0.0341 (13)	0.0394 (14)	0.0340 (13)	−0.0008 (10)	−0.0004 (10)	0.0005 (10)

### Geometric parameters (Å, º)

**Table:**

C1—O1	1.262 (3)	O4—C11	1.417 (3)
C1—C2	1.448 (3)	C11—H11A	0.9800
C1—C13	1.470 (3)	C11—H11B	0.9800
C2—C3	1.347 (3)	C11—H11C	0.9800
C2—H2	0.9500	C12—H12	0.9500
C3—C4	1.437 (3)	C13—C14	1.379 (3)
C3—H3	0.9500	C13—C22	1.436 (3)
C4—C5	1.406 (3)	C14—O5	1.352 (3)
C4—C12	1.418 (3)	C14—C15	1.433 (3)
C5—O2	1.376 (3)	O5—H5	0.8400
C5—C7	1.381 (3)	C15—C16	1.401 (3)
O2—C6	1.421 (3)	C15—C20	1.422 (3)
C6—H6A	0.9800	C16—C17	1.376 (4)
C6—H6B	0.9800	C16—H16	0.9500
C6—H6C	0.9800	C17—C18	1.400 (4)
C7—C8	1.383 (3)	C17—H17	0.9500
C7—H7	0.9500	C18—C19	1.362 (4)
C8—O3	1.349 (3)	C18—H18	0.9500
C8—C10	1.416 (3)	C19—C20	1.419 (3)
O3—C9	1.432 (3)	C19—H19	0.9500
C9—H9A	0.9800	C20—C21	1.412 (3)
C9—H9B	0.9800	C21—C22	1.358 (3)
C9—H9C	0.9800	C21—H21	0.9500
C10—C12	1.361 (3)	C22—H22	0.9500
C10—O4	1.378 (3)		
			
O1—C1—C2	119.8 (2)	O4—C11—H11B	109.5
O1—C1—C13	118.6 (2)	H11A—C11—H11B	109.5
C2—C1—C13	121.6 (2)	O4—C11—H11C	109.5
C3—C2—C1	121.5 (2)	H11A—C11—H11C	109.5
C3—C2—H2	119.3	H11B—C11—H11C	109.5
C1—C2—H2	119.3	C10—C12—C4	121.9 (2)
C2—C3—C4	128.5 (2)	C10—C12—H12	119.1
C2—C3—H3	115.8	C4—C12—H12	119.1
C4—C3—H3	115.8	C14—C13—C22	118.1 (2)
C5—C4—C12	117.1 (2)	C14—C13—C1	120.3 (2)
C5—C4—C3	120.1 (2)	C22—C13—C1	121.6 (2)
C12—C4—C3	122.8 (2)	O5—C14—C13	121.7 (2)
O2—C5—C7	123.0 (2)	O5—C14—C15	116.21 (19)
O2—C5—C4	115.6 (2)	C13—C14—C15	122.0 (2)
C7—C5—C4	121.4 (2)	C14—O5—H5	109.5
C5—O2—C6	117.6 (2)	C16—C15—C20	119.9 (2)
O2—C6—H6A	109.5	C16—C15—C14	122.3 (2)
O2—C6—H6B	109.5	C20—C15—C14	117.8 (2)
H6A—C6—H6B	109.5	C17—C16—C15	120.7 (2)
O2—C6—H6C	109.5	C17—C16—H16	119.6
H6A—C6—H6C	109.5	C15—C16—H16	119.6
H6B—C6—H6C	109.5	C16—C17—C18	119.7 (2)
C5—C7—C8	120.3 (2)	C16—C17—H17	120.1
C5—C7—H7	119.9	C18—C17—H17	120.1
C8—C7—H7	119.9	C19—C18—C17	120.9 (2)
O3—C8—C7	125.1 (2)	C19—C18—H18	119.6
O3—C8—C10	115.3 (2)	C17—C18—H18	119.6
C7—C8—C10	119.6 (2)	C18—C19—C20	121.1 (2)
C8—O3—C9	117.9 (2)	C18—C19—H19	119.4
O3—C9—H9A	109.5	C20—C19—H19	119.4
O3—C9—H9B	109.5	C21—C20—C19	122.4 (2)
H9A—C9—H9B	109.5	C21—C20—C15	119.9 (2)
O3—C9—H9C	109.5	C19—C20—C15	117.7 (2)
H9A—C9—H9C	109.5	C22—C21—C20	120.7 (2)
H9B—C9—H9C	109.5	C22—C21—H21	119.7
C12—C10—O4	126.2 (2)	C20—C21—H21	119.7
C12—C10—C8	119.7 (2)	C21—C22—C13	121.5 (2)
O4—C10—C8	114.1 (2)	C21—C22—H22	119.2
C10—O4—C11	116.46 (19)	C13—C22—H22	119.2
O4—C11—H11A	109.5		
			
O1—C1—C2—C3	4.1 (4)	C2—C1—C13—C14	−177.7 (2)
C13—C1—C2—C3	−175.7 (2)	O1—C1—C13—C22	−177.4 (2)
C1—C2—C3—C4	−179.1 (2)	C2—C1—C13—C22	2.5 (3)
C2—C3—C4—C5	−176.7 (2)	C22—C13—C14—O5	179.5 (2)
C2—C3—C4—C12	4.6 (4)	C1—C13—C14—O5	−0.3 (3)
C12—C4—C5—O2	−178.9 (2)	C22—C13—C14—C15	−0.3 (3)
C3—C4—C5—O2	2.3 (3)	C1—C13—C14—C15	179.9 (2)
C12—C4—C5—C7	1.4 (3)	O5—C14—C15—C16	−0.6 (3)
C3—C4—C5—C7	−177.4 (2)	C13—C14—C15—C16	179.2 (2)
C7—C5—O2—C6	−6.7 (4)	O5—C14—C15—C20	−179.3 (2)
C4—C5—O2—C6	173.6 (2)	C13—C14—C15—C20	0.5 (3)
O2—C5—C7—C8	179.6 (2)	C20—C15—C16—C17	0.8 (4)
C4—C5—C7—C8	−0.7 (4)	C14—C15—C16—C17	−177.9 (2)
C5—C7—C8—O3	178.0 (2)	C15—C16—C17—C18	−0.6 (4)
C5—C7—C8—C10	−0.3 (4)	C16—C17—C18—C19	−0.3 (4)
C7—C8—O3—C9	−3.4 (4)	C17—C18—C19—C20	1.1 (4)
C10—C8—O3—C9	174.9 (2)	C18—C19—C20—C21	178.1 (3)
O3—C8—C10—C12	−177.8 (2)	C18—C19—C20—C15	−1.0 (4)
C7—C8—C10—C12	0.6 (4)	C16—C15—C20—C21	−179.0 (2)
O3—C8—C10—O4	0.5 (3)	C14—C15—C20—C21	−0.3 (3)
C7—C8—C10—O4	178.9 (2)	C16—C15—C20—C19	0.0 (3)
C12—C10—O4—C11	−6.0 (4)	C14—C15—C20—C19	178.8 (2)
C8—C10—O4—C11	175.8 (2)	C19—C20—C21—C22	−179.1 (2)
O4—C10—C12—C4	−178.0 (2)	C15—C20—C21—C22	−0.1 (4)
C8—C10—C12—C4	0.1 (4)	C20—C21—C22—C13	0.2 (4)
C5—C4—C12—C10	−1.1 (3)	C14—C13—C22—C21	−0.1 (4)
C3—C4—C12—C10	177.6 (2)	C1—C13—C22—C21	179.7 (2)
O1—C1—C13—C14	2.4 (3)		

### Hydrogen-bond geometry (Å, º)

**Table:**

*D*—H···*A*	*D*—H	H···*A*	*D*···*A*	*D*—H···*A*
O5—H5···O1	0.84	1.74	2.490 (2)	147
C21—H21···O3^i^	0.95	2.43	3.362 (3)	166

Symmetry code: (i) −*x*+3/2, *y*−1/2, −*z*+1/2.
